# Hemagglutination inhibiting antibodies and protection against seasonal and pandemic influenza infection

**DOI:** 10.1016/j.jinf.2014.09.003

**Published:** 2015-02

**Authors:** Annette Fox, Le Quynh Mai, Le Thi Thanh, Marcel Wolbers, Nguyen Le Khanh Hang, Pham Quang Thai, Nguyen Thi Thu Yen, Le Nguyen Minh Hoa, Juliet E. Bryant, Tran Nhu Duong, Dang Dinh Thoang, Ian G. Barr, Heiman Wertheim, Jeremy Farrar, Nguyen Tran Hien, Peter Horby

**Affiliations:** a Oxford University Clinical Research Unit and Wellcome Trust Major Overseas Programme, Viet Nam; b Center for Tropical Medicine, Nuffield Department of Clinical Medicine, University of Oxford, Oxford, UK; c The University of Melbourne, Peter Doherty Institute for Infection and Immunity, Department of Microbiology and Immunology, Parkville, Victoria, Australia; d National Institute of Hygiene and Epidemiology, Hanoi, Viet Nam; e Ha Nam Centre for Preventive Medicine, Ha Nam, Viet Nam; f World Health Organization Collaborating Centre for Reference and Research on Influenza, Victorian Infectious Diseases Reference Laboratory, North Melbourne, Australia

**Keywords:** Influenza, Human, Hemagglutination inhibition tests, Immunity, Humoral, Antibody, Neutralizing, Pandemics, Humans

## Abstract

**Objectives:**

Hemagglutination inhibiting (HI) antibodies correlate with influenza vaccine protection but their association with protection induced by natural infection has received less attention and was studied here.

**Methods:**

940 people from 270 unvaccinated households participated in active ILI surveillance spanning 3 influenza seasons. At least 494 provided paired blood samples spanning each season. Influenza infection was confirmed by RT-PCR on nose/throat swabs or serum HI assay conversion.

**Results:**

Pre-season homologous HI titer was associated with a significantly reduced risk of infection for H3N2 (OR 0.61, 95%CI 0.44–0.84) and B (0.65, 95%CI 0.54–0.80) strains, but not H1N1 strains, whether re-circulated (OR 0.90, 95%CI 0.71–1.15), new seasonal (OR 0.86, 95%CI 0.54–1.36) or pandemic H1N1-2009 (OR 0.77, 95%CI 0.40–1.49). The risk of seasonal and pandemic H1N1 decreased with increasing age (both *p* < 0.0001), and the risk of pandemic H1N1 decreased with prior seasonal H1N1 (OR 0.23, 95%CI 0.08–0.62) without inducing measurable A/California/04/2009-like titers.

**Conclusions:**

While H1N1 immunity was apparent with increasing age and prior infection, the effect of pre-season HI titer was at best small, and weak for H1N1 compared to H3N2 and B. Antibodies targeting non-HI epitopes may have been more important mediators of infection-neutralizing immunity for H1N1 compared to other subtypes in this setting.

## Highlights

• The determinants of influenza immunity were examined in an unvaccinated cohort.
• The risk of H3N2 and B infection decreased with increasing pre-season HI titer.
• Pre-season HI titer had less effect on H1N1 infection.
• H1N1 immunity increased with age and seasonal H1N1 induced pandemic H1N1 immunity.
• The contribution of non-HI antibodies to immunity may be relatively high for H1N1.

## Background

Each year, seasonal influenza is responsible for three to five million severe illnesses and 250,000 to 500,000 deaths worldwide. An accurate and complete understanding of the mechanisms of immunity to influenza is critical in order to assess the risk posed by new virus variants and to optimize immunization strategies. Influenza viruses infect human cells through the binding of the viral surface hemagglutinin (HA) protein to the terminal sialic acid molecules of glycoproteins and glycolipids expressed on host cell membranes, and the subsequent fusion of viral and cell membranes.^1^ Antibodies directed at targets surrounding the receptor-binding pocket of the HA can block binding, and are the best-defined correlate of influenza immunity. Serum concentrations of antibodies that block receptor binding are traditionally measured using the hemagglutination inhibition (HI) assay, and HI titers of between 18 and 40 are associated with a 50% reduction in infection risk.^2–8^ However, the determinants of immunity to influenza in humans remain incompletely understood, with HI antibodies providing only a partial explanation. Indeed, in his seminal paper describing the protective effect of pre-existing HI antibodies on H3N2 and B infection, Hobson noted that people with no detectable HI antibodies may be resistant to infection,^3^ and it is well recognized that immunity to infection can span major antigenic variants within a subtype.^9–13^ When H1N1 re-emerged in 1977 after an absence of 20 years, resistance to infection in people aged over 20 years was not dependent on HI antibodies^6,10^ and in 2009, adults in several Asian countries experienced low rates of pandemic H1N1 infection despite the virtual absence of detectable homologous HI antibodies.^12–16^

Influenza viruses have a high potential for genetic and antigenic diversity, and influenza epidemiology is characterized by regular epidemics of antigenically distinct strains.^17^ Since the binding region of the HA1 protein is a key target for neutralising antibodies, it is under intense immune-mediated positive selection pressure, resulting in the acquisition and retention of amino acid substitutions that favor escape from immunity. However, the rate of antigenic evolution of the HA1 differs between subtypes, with H3N2 evolving faster than H1N1,^18,19^ an observation for which there is considerable uncertainty over the mechanisms underlying this difference.^20^

We set out to re-examine the contribution of serum HI antibody to protection against natural influenza infection in an unvaccinated Vietnamese cohort followed over three consecutive influenza transmission periods, which included re-circulating strains, new antigenic variants, and the first wave of the 2009 H1N1 pandemic.

## Participants

The research was approved by the institutional review board of the National Institute of Hygiene and Epidemiology, Vietnam; the Oxford Tropical Research Ethics Committee, University of Oxford, UK; and the Ethics Committee of the London School of Hygiene and Tropical Medicine, UK. All participants provided written informed consent.

The procedures for selecting the study site and for selecting and investigating individual participants are described in detail elsewhere.^21^ In brief, households in Thanh Ha commune, Thanh Liem District, Ha Nam Province, Viet Nam were selected at random. This semi-rural commune is in the Red-River delta around 60 km from Hanoi. 940 members of 270 randomly selected households consented and were enrolled. The cohort is ongoing but the analysis described here covers three consecutive influenza seasons detected up until April 2010 (Table 1). Influenza seasons were detected via active surveillance for influenza-like-illness (ILI), defined as a fever > 38 °C and cough or sore throat. Study health workers examined participants with ILI and collected nose and throat swabs. Investigation was enhanced during the first wave of pandemic H1N1 transmission (September–December 2009) when all members of ILI case households were swabbed daily for up to 15 days. Blood samples were collected for serology at baseline in December 2007 and between each confirmed influenza season (Table 1).

## Methods

### Virology and serology

Combined nose and throat swabs were assessed by real-time reverse-transcriptase polymerase chain reaction (RT-PCR), according to WHO/US CDC protocols (CDC reference no. I-007-05, Accessed November 30, 2009, at http://www.who.int/csr/resources/publications/swineflu/CDCRealtimeRTPCR_SwineH1Assay-2009_20090430.pdf). Viruses were isolated from participants' swabs and propagated in MDCK cells. The HA genes of seasonal H1N1 and H3N2 isolates were amplified and DNA sequencing performed using a 3100 genetic analyzer and BigDye Terminator Mix v3.0 (Applied Biosystems Inc.). Genome sequences representing vaccine strains and some with >93% identity to isolates sequenced in this study were downloaded from the NCBI Influenza Virus Resource (http://www.ncbi.nlm.nih.gov/genomes/FLU/FLU.html). Alignment of multiple sequences was performed by the ClustalW method.^22^ Phylogenetic trees were constructed using the maximum likelihood and neighbor-joining methods in the PHYLIP software package (version 3.66, University of Washington, Seattle, WA).^23^ Seasonal H3N2 and B isolates also underwent thorough antigenic characterization by the WHO Collaborating Center for Reference and Research in Influenza in Melbourne, Australia. One H1N1 isolate from 2008 to 2 from 2009 were assessed in HI assay with seasonal H1N1 reference sera provided in the 2010–2011 WHO Influenza Reagent Kit For Identification of Influenza Isolates (produced and distributed by: WHO Collaborating Center for Surveillance, Epidemiology and Control of Influenza, Centers for Disease Control and Prevention, Atlanta, Georgia 30333, U.S.A).

Venous blood was collected into heparin vacutainers for the first two collection times and into serum vacutainers for the last two collection times. Plasma or sera was separated within 4 h and stored at −20 °C. Paired plasma/sera were tested in hemagglutination inhibition (HI) assay as previously described.^21^ Seasonal influenza H1N1 and H3N2 viruses isolated from participants' swabs and propagated in MDCK cells were used for HI assay with serum pairs spanning season 1. The same H1N1 virus was used to assess season 2 plasma whereas the H3N2 virus used (TX265) was isolated from a patient presenting in Hanoi in the same season, and propagated in embryonated hen's eggs. This virus was genetically and antigenically similar to viruses isolated from participants' swabs (Supplementary Information). A single influenza B virus isolated from a participant during 2008, and propagated in MDCK cells was used to assess serum for both the first and second seasons. The virus had a titer of 320 with B/Wisconsin/1/2010 (Yamagata) reference antisera and of <10 with B/Brisbane/60/2008 (Victoria) antisera. A reference antigen supplied by WHO (A/California/7/2009(H1N1)-like) was used to assess season 3/pandemic plasma. The HI titer was read as the reciprocal of the highest serum dilution causing complete inhibition of agglutination, partial agglutination was not scored as inhibition of agglutination. If there was no inhibition of HI at the highest serum concentration (1:10 dilution) the titer was designated as 5. Only one sample had a titer >1280 and this was not adjusted.

### Definitions and analysis

‘Influenza infection’ was defined as either the detection of influenza RNA in a swab sample by RT-PCR or a four fold or greater rise in HI titer, with a second titer of at least 40.

Participants were excluded from analysis of each season if they were not present for ILI surveillance during the periods of confirmed influenza transmission or if paired-plasma were not collected. Additionally, participants were excluded from the analysis of effect of infection in one season on infection in subsequent season if they had not been available or fully assessed for infection in both seasons.

The risk of an infection was modeled as depending on the (log2-transformed) pre-season titer using a marginal logistic regression model, which takes into account potential household clustering. Unadjusted titer effects and titer effects adjusted for age (modeled as a natural cubic spline with 3 degrees of freedom and knots at 10 and 20 years) were calculated. We also tested for potential non-linear effects of the log2-titer on outcome by additionally including a quadratic term into the model and for titer–age interactions. The risk of infection was also modeled as depending on infection in the preceding season with each strain that did not induce HI antibodies (i.e. prior heterologous infections). As above, marginal logistic regression was used to account for potential household clustering and results adjusted for effects of age and pre-season HI titer. Statistical analyses were performed with the statistical software R version 2.15.0 (R foundation for Statistical Computing, Vienna, Austria) and the companion R package geepack version 1.1-6.

## Results

### Participants and infections

A detailed description of the cohort and of the infections and illnesses detected has been presented previously.^21^ In brief, 940 individuals were studied for three consecutive influenza seasons, from December 2007 through April 2010, resulting in 1793 person-seasons of influenza surveillance. The age of participants ranged from <1 to 90 years and none had ever received influenza vaccination. Weekly active surveillance for episodes of influenza-like illness (ILI) was conducted, and a nose swab and throat swab obtained from ILI cases for the detection of influenza RNA by reverse transcription polymerase chain reaction (RT-PCR). Participants who were 5 years of age or older at the time of sampling were asked to provide blood at recruitment and after each peak in confirmed case detection for paired serology. Age- and sex-standardized estimates of the risk of influenza infection and illness per season in persons 5 years of age or older were reported previously.^21^

**Fig. S1:**
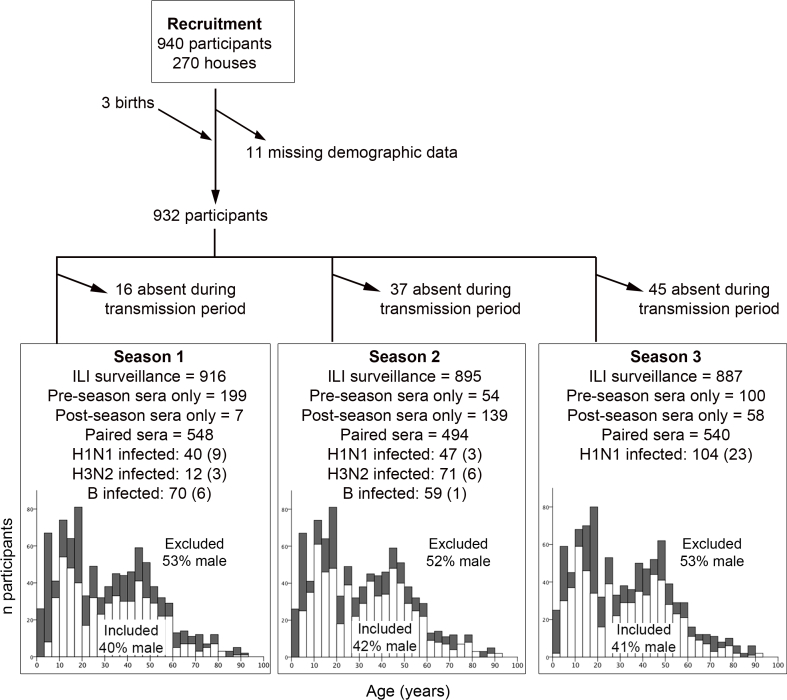
Selection of participants for analysis and number analyzed that were infected. Numbers in parentheses show the numbers of infections that were RT-PCR confirmed.

**Fig. S2:**
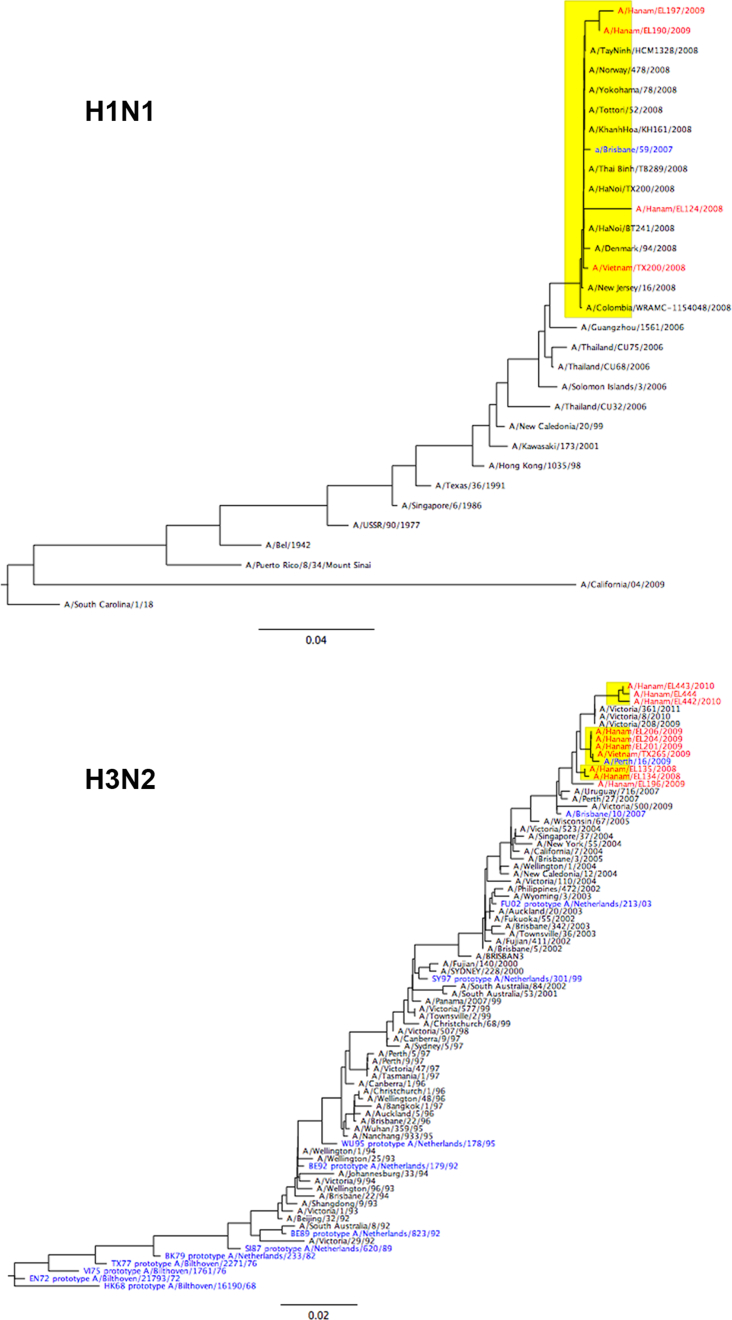
Phylogenetic analysis of the HA genes of H3N2 and H1N1 viruses isolated from cohort participants (Shown in red) in 2008 (S1) and 2009 (S2). H3N2 HA sequences were provided by the WHO Collaborating Centre for Reference and Research on Influenza, VIDRL, as part of the Global Influenza Surveillance and Response System. Vaccine/reference strains are shown in blue.

Three influenza seasons were identified in this study period (Table 1). The number of people that provided blood samples spanning each season, the numbers infected as determined by serology and RT-PCR, and their age distribution is shown as supplementary information (Fig. S1). Males, and participants aged less than 5 or in their late teens were under-represented in the group that could be analyzed (Fig. S1). Genetic and antigenic characterization of the viruses isolated and used for serology is shown in supplementary information (Fig. S2 and Table S1). The H1N1 viruses isolated in season one (S1) in 2008 were A/Brisbane/59/2007-like, and B virus isolates were of the B-Yamagata-lineage and were B/Florida/4/2006-like, representing strains that were antigenically distinct from the pre-study season. The H3N2 viruses isolated in S1 were antigenically A/Brisbane/10/2007-like, as in the pre-study season, and caused few infections. The H3N2 viruses isolated in the second season (S2) in Spring 2009 were antigenically distinct A/Perth/16/2009-like strains, and caused the highest incidence of infection, whereas two H1N1 isolates were similar to the S1 isolate. HI titers with WHO reference sera against seasonal H1N1 were 1280 against the 2008 H1N1 isolate and 640 against both 2009 H1N1 isolates. The only B virus isolated in 2009 belonged to the B-Victoria lineage, and the National Influenza surveillance system identified a shift in B-lineage predominance from Yamagata to Victoria in 2009. However serology was only performed with a Yamagata lineage virus. The third season (S3) in Autumn 2009, was caused by the pandemic H1N1 2009 strain (A/California/04/2009), which resulted in a high incidence of infection compared to individual seasonal strains.

It was not feasible to collect swabs from all cohort participants weekly; hence infections were also identified by HI antibody seroconversion. As in our previous report, seroconversion was defined as at least a 4-fold rise in titer with a post-season titer of at least 40.^21^ We have recently reported that the pattern of 2-fold increases in HI titer cannot be fully explained by assay variability, and that a reliance on four-fold titer increases to define infection may under estimate the true incidence of infection.^24^ However, since it is not possible to adjust for assay variability in an individual level analysis we did not apply a 2-fold definition.

The seroconversion rate amongst virologically confirmed cases indicated that the sensitivity of serology was high for detecting H3N2 infection (9/9, 100%), but only 66% (23/35) for H1N1 infection (Supplementary, Table S2). Post-infection geometric mean HI titers were significantly higher for virologically confirmed H3N2 cases compared to H1N1 cases (*p* < 0.001) with values of 218 (95%CI 113–421) and 40 (95%CI 26–62), respectively. A number of participants with virologically confirmed H1N1 that did not seroconvert, according to our pre-defined criteria, exhibited a 2-fold increase in titer or a 4-fold increase from 5 to 20.

### Detection of HI antibody in pre-season plasma and effect on homosubtypic infection

The proportion of participants with HI antibody titers of 20 or more in pre-season plasma ranged between 11% and 48% for seasonal influenza strains but was only 2.3% for pandemic A/California/04/2009-like virus.

The effect of pre-season serum/plasma HI titer on subsequent homosubtypic infection was investigated for each subtype and season. Log2 titers were modeled to affect the log-odds of the risk of infection linearly with adjustment for age (Table 2). There was a significant linear effect of HI titer on the risk of infection for H3N2 in S2 and influenza B (Yamagata lineage) in S1 and S2 but not for H1N1 in S1, S2 or S3. There was no evidence for a non-linear (quadratic) association for any of the analyses (all *p* > 0.1), except for H1N1 in S2 (*p* = 0.01), where there was evidence that titers ≥ 80 may decrease the risk of infection.

After adjusting for HI titer, age was independently associated with decreasing risk of infection for H1N1 in S1 (*p* = 0.08), S2 (*p* < 0.0001), and pandemic S3 (*p* < 0.0001) and for H3N2 in S2 (*p* = 0.03), however there was no significant age effect for influenza B (Yamagata lineage) (*p* > 0.6 in S1 and S2). This is concordant with age effects, unadjusted for titer, discussed in detail in our previous report.^21^ There was no evidence for titer–age interactions (all *p* > 0.3), except for H3N2 in S1 (*p* = 0.06).

To examine whether the relation between HI titer and protection is significantly different for H1N1 compared to H3N2 and B, the association between infection with a strain and the HI titer against that strain was modeled with an interaction with other strains. The effect of HI titer was significantly different for H3N2 and B versus H1N1, but this was mainly due to differences during season 2 (Table 2).

The effect of including titer rises from 5 (<10) to 20 in the definition of seroconversion and hence infection was examined (Supplementary, Table S3). All associations that were significant using the original definition of infection remained significant. In addition, unadjusted and age-adjusted associations between pre-season H3N2 titer and infection in season 1 were significant with the new definition, and other significant effect sizes were greater, reflecting increases in the numbers defined as infected amongst participants whose pre-season titer was 5. The effects of pre-season titer on homologous H1N1 strain infection remained insignificant with the exception of H1N1 in S2, which was significant only after adjusting for age. There was no evidence for titer–age interactions.

**Fig. S3:**
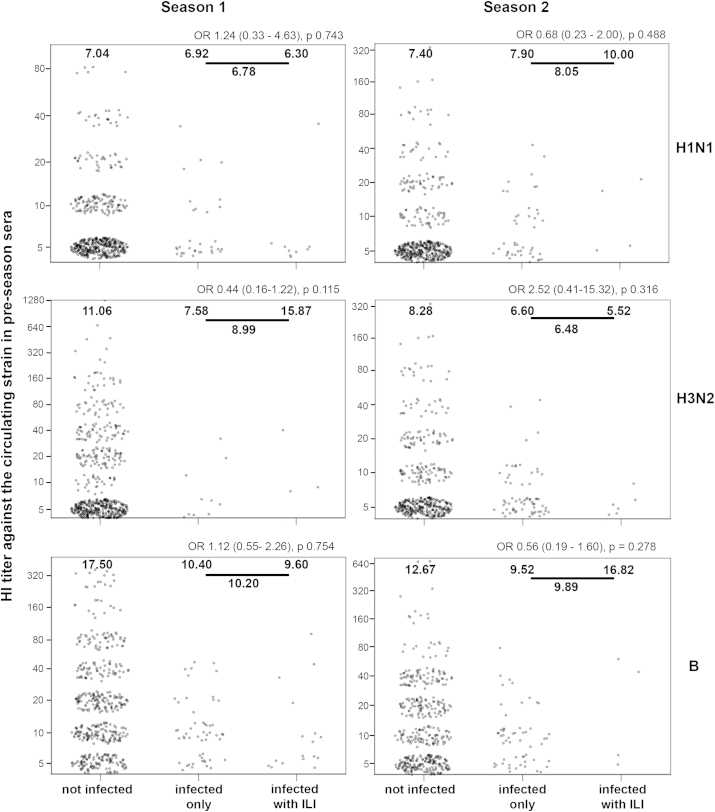
Association between pre-season HI titer and infection and illness status for each subtype in season 1 and 2. Each plot shows HI titers, which fall into up to nine discrete values but have been dispersed for visualization. Geometric mean titers are shown within the plots for each group and for the combined infected groups. Odds ratios with confidence intervals and p values for the association between pre-season titer and influenza-like-illness (ILI) development amongst infected participants are shown above each plot.

The number of participants with ILI confirmed as influenza was small (Fig. S1), and associations between HI titer and illness amongst those infected were not significant, although there was trend for participants who developed ILI after H3N2 infection in season 2 to have lower pre-season titers (Fig. S3).

### Effect of prior heterologous infection

To further investigate whether non-HI antibodies contribute to protection against infection we assessed the effect of infection in S1 or S2 on infection in S2 or S3 respectively, when the first infection did not induce HI antibodies to the second infection (Table 3). This analysis was limited to comparisons across different subtypes with the exception of H1N1 in S2, which was not associated with production of HI antibodies to pandemic H1N1 in S3 (*p* = 0.921). Associations between influenza A and B infections were investigated to verify whether effects reflect adaptive antibody responses as opposed to non-specific mechanisms. For S2, there was no detectable effect of prior H1N1 infection on subsequent H3N2 infection or vice versa but the numbers infected were small and confidence intervals were large, particularly for the effects of H3N2. However, infection with H1N1 in S2 was associated with a clear reduction in the risk of pandemic H1N1 infection in S3, whereas B (Yamagata) had the opposite effect and H3N2 had no significant effect. There was no similar effect of B in S1 on H1N1 in S2 despite similar sample sizes. The effects of H1N1 and B infection in S2 on pandemic H1N1 infection in S3 were maintained after adjusting for age and pre-season HI titer, and when both prior H1N1 and B were included together in the same model.

## Discussion

In subjects whose influenza immunity has been shaped by prior natural infection without vaccination, protection against infection was significantly associated with homologous HI titer for H3N2 and B Yamagata lineage but not for H1N1. However, protection against H1N1 infection was associated with increasing age, and protection against pandemic H1N1 was also associated with prior confirmed seasonal H1N1 infection, even though HI antibodies were rarely detected. It was also clear that HI antibodies were not always induced following H1N1 infection and titers induced were low relative to H3N2 infection. The lower levels of H1N1 HI seroconversion following virologically confirmed infection means that we may have underestimated the proportion of participants that were H1N1 infected, and this potential under-ascertainment of infections would be concentrated amongst those with low baseline titers. This could be one factor that decreases the likelihood of detecting a significant protective effect of H1N1 HI titers, but also indicates a difference between the subtypes with respect to HI antibody. There was some indication that high HI titers (≥80) protected against H1N1 in season 2, whereas results for linear effects of HI antibodies on H1N1 were consistent for three transmission periods including a re-circulated, a newly drifted, and a shifted strain. Confidence intervals, especially for season 2, were sufficiently narrow to rule out a large linear effect of HI titer.

The findings presented here must be reconciled with the long-standing view that HI titers correlate with protection,^3,5,25–27^ and with evidence that HI antibodies block virus binding to host cell membrane receptors, correlate with neutralization in tissue and egg culture, and transfer protection in mice.^3,5,28^ An important factor to consider is that the challenge studies that first established a correlation between HI titer and protection did not include H1N1 strains^3,27,29^ and many subsequent studies have looked at HI antibodies induced by inactivated subunit vaccines given intramuscularly rather than by natural infection via the respiratory route.^7,25,26,30^ There is substantial evidence that inactivated vaccine and live virus infections induce different antibodies. It is particularly well established that intranasal live attenuated influenza vaccines (LAIV) provide equivalent protection to inactivated vaccine although HI titers are invariably lower and underestimate efficacy.^7,29,31–35^ Neutralizing antibody titer, influenza specific airway IgA, influenza specific IgG + B cell frequency, or combinations of these factors correlate better with LAIV efficacy.^33–36^ Nevertheless, a number of natural infection cohorts have demonstrated correlations between homologous HI titers and protection against H1N1 infection. In some of these studies participants had very little prior exposure to natural H1N1 infection, as in a study of boarding school students just 3 years after H1N1 re-emerged.^37^ In others immunity may have been shaped by vaccination, as in two cohort studies that enrolled adults soon after the 2009 pandemic started.^38,39^ At least 10% of participants in these cohorts had received seasonal influenza vaccine, and the proportions with detectable pandemic H1N1 HI antibody at baseline was at least 2-fold higher than in the present study. Another study found a significant effect of baseline titer on pandemic H1N1 infection in adults that had not had influenza vaccine in the preceding season of whom 10–15% had already been infected at baseline.^40^ In the present study cohort participants had never been vaccinated against influenza, and only 6% had a detectable pandemic H1N1 antibody titer at baseline, most of whom has titers of just 10. This indicates that the association between HI antibodies and protection against H1N1 may vary depending on the population or strains involved and timing of investigation in relation to antigenic drift or shift.

Numerous other studies of the 1977 and 2009 H1N1 pandemics found that infection risk was associated with age independent of HI antibody titers, and suggest that this phenomenon is due to broadly neutralizing, non-HI antibodies.^6,11–13,38,39,41–43^ We therefore reconcile our results by hypothesizing that while HI antibodies can neutralize H1N1 virus and provide immunity against infection, they are not always the only or indeed the dominant type of antibody mediating this protection.

This study has several limitations. We do not know how HI titers in pre-season plasma relate to titers at the time of influenza transmission because HI titers decay, particularly in the first six months after infection.^10^ We have previously reported that HI titer decay was most common during the first season when the interval between pre- and post-season sample collection was longest.^24^ Over this season H3N2 titers decayed in 30% of participants and B titers in 11%, consistent with circulation of these strains just prior to collection of baseline plasma. In contrast, H1N1 HI titers decayed in only 1% of participants during each of the 3 seasons assessed.^24^ Therefore antibody titer decay cannot explain the observed differences between H1N1, H3N2, and B. We cannot rule out the possibility that HA-directed antibodies that block H1N1 virus binding to respiratory epithelial cells are present but not detected by the HI assay with red blood cells. However, results were consistent for two different H1N1 and H3N2 strains; all HI assays were performed using the same protocol and for season 2 all tests were performed with the same batch of red blood cells; and our protocol was validated by testing subsets of sera in other internal and external laboratories. HI titers in serum and plasma correlate well with more than 80% agreement for seroconversion, but plasma titers are lower.^44^ Therefore, pre-season 1 and 2 titers may be underestimated, but effects will be the same across subtypes. Although we did not find a significant effect of baseline HI titer on H3N2 infection during season 1, there were a very small number of H3N2 infections in that season (*n* = 12) and effects were significant if we expanded the definition of infection to include four-fold changes in antibody level from titer 5 to 20. Finally, we did not perform serology to identify B Victoria lineage infections so do not know if there was an effect of HI titer on infection for this lineage. It will be important to examine effects of past infection with one lineage on infection with the other lineage in future.

Our findings indicate that in this unvaccinated population prior natural influenza H1N1 infections induced immunity against infection with new drifted and novel strains, which did not appear to be reliant on HI antibodies. Further, this putative non-HI neutralizing activity may be a predominant source of H1N1 neutralization. A similar inference was drawn from the English physicians study (1973–1978), which concluded that “factors other than strain-specific antibodies may be responsible in protecting against influenza during a period of drift”.^45^ In ferrets, infection with a sequence of antigenically distinct seasonal H1N1 viruses elicits antibodies that protect against novel 2009 H1N1, whereas no single seasonal H1N1 virus assessed elicited cross-protective antibody.^46^ Ferret studies using shifted challenge strains may help to determine whether the breadth of protection, or cross-neutralization, induced by sequential variant strain infections is greater for H1N1 than for H3N2. Repeated infection with different live virus strains preferentially induces HA cross-reactive antibodies,^10^ and we hypothesize that these include pan-H1N1 neutralizing antibodies. One of the best-described targets for cross-neutralizing antibodies is the membrane-proximal region of HA that facilitates fusion; this region is conserved amongst H1N1 strains but distinct from H3N2.^1,47^ Antibodies that inhibit fusion are technically difficult to detect,^48^ but have been found amongst broadly-neutralizing monoclonal antibodies raised in mice^49,50^ and in human phage-display antibody libraries.^1,47,51–53^ It will also be important to examine neuraminidase inhibiting (NI) antibodies, which have been associated with protection against both infection and illness independent of effects of HI antibodies.^40^ Recent studies also describe the detection of cross-reactive antibodies that trigger NK cell activation and *in vitro* elimination of influenza-infected cells in people lacking HI antibodies.^54^

If the phenomenon observed in this study is replicable and widespread it may account for differences in the rate of antigenic evolution of the HA1 region of H1N1 compared to H3N2, as evidenced by nineteen drift variants identified for H3N2 over a 29 year period but only 6 for H1N1.^18^ Specifically, if the contribution of HI antibodies relative to non-HI antibodies to virus neutralization is less for H1N1 than for H3N2, then the selective advantage of mutations within HI antibody binding sites will be less, and antigenic evolution will be slower. This hypothesis is consistent with the lower post-infection geometric mean HI titers we observed amongst RT-PCR confirmed H1N1 cases compared to H3N2 cases, with similar findings reported for the comparison of live attenuated H1N1 and H3N2 vaccines^55^ and for studies of vaccine responses in the elderly.^56^ Non-HI antibodies could prevent HI antibody induction either by enhancing virus clearance or by competing for antigen. It will be important to confirm whether non-HI neutralizing antibodies account for the absence of a detectable protective effect of baseline H1N1 HI antibodies in our cohort.

## Funding

This work was supported by the Wellcome Trust UK (grants 081613/Z/06/Z; 077078/Z/05/Z; and 087982AIA). AF was supported by the European Union FP7 project ‘‘European Management Platform for Emerging and Re-emerging Infectious Disease Entities (EMPERIE)’’ (no. 223498).

## Figures and Tables

**Table 1:** Timing and intensity of influenza transmission in the cohort.

Period	Year (months) season^a^	Subtype	Strain^b^	% infected(95% CI)^c^
Pre-study	2007	H1N1	Solomon Islands/3/06-like	–
	2007	H3N2	Brisbane/10/2007-like	–
	2007	B	Yamagata Florida/04/2006-like	–
Bleed 1	2007 (12) Winter			
Season 1	2008 (7–8) Summer	H1N1	**Brisbane/59/2007-like**	7.2 (5.3–9.7)
	2008 (8–9) Autumn	H3N2	Brisbane/10/2007-like	2.3 (1.3–4.0)
	2008 (2,4,6,9,10,12)	B	**Yamagata Florida/04/2006-like**^d^^,^^e^	12.6 (10.1–15.6)
Bleed 2	2008 (12) Winter			
Season 2	2009 (4) Spring	H1N1	Brisbane/59/2007-like	8.6 (6.6–11.0)
	2009 (4–6) Spring	H3N2	**Perth/16/2009-like**	13.1 (10.7–16.0)
	2009 (4) Spring	B	Victoria Cambodia/30/2011-like^e^	10.5 (8.3–13.1)
Bleed 3	2009 (6) Summer			
Season 3	2009 (9–12) Autumn	H1N1	**California/04/2009-like**	18.2 (15.3–21.5)
Bleed 4	2010 (4) Spring			

a Months (January = 1 – December = 12) when influenza virus RNA was detected in swabs by RT-PCR.

b Strain designation is based on National Influenza Surveillance programme data from Northern Vietnam coordinated by the National Influenza Center at the National Institute of Hygiene and Epidemiology. Strains considered to be antigenically distinct compared to those circulating previously are shown in bold.

c The number of participants assessed is shown in Table 2.

d This strain started to circulate just prior to study commencement and continued to circulate during the study.

e Six influenza B strains were isolated in S1 and belonged to the Yamagata lineage whereas a single strain was isolated in S2 and belonged to the Victoria lineage (Table S1).

**Table 2:** Pre-season HI titer detection and effect on homosubtypic infection.

Season	Infecting/serology strain	Pre-season HI titer	*n* infected/n with titer (%)	OR for each 2-fold titer increase [95% CI]	OR for each 2-fold titer increase, adjusted for age [95% CI]
1	H1N1	<=10	35/486 (7.2)	0.95 [0.63–1.43];	0.86 [0.54–1.36];
	A/Brisbane/59/2007-like	20	3/36 (8.3)	*p* = 0.79	*p* = 0.52
		40	2/21 (9.5)		
		80	0/5 (0.0)		
1	H3N2	<=10	9/359 (2.5)	0.91 [0.71–1.17];	0.75 [0.48–1.16];
	A/Brisbane/10/2007-like	20	1/73 (1.4)	*p* = 0.45	*p* = 0.19
		40	2/56 (3.6)		
		≥80	0/60 (0.0)		
1	B/Florida/04/2006-like	<=10	53/285 (18.6)	0.65 [0.53–0.79];	0.65 [0.54–0.80];
		20	8/121 (6.6)	*p* < 0.0001	*p* < 0.0001
		40	8/67 (11.9)		
		≥80	1/75 (1.3)		
2	H1N1	<=10	38/410 (9.3)	1.10 [0.89–1.36];	0.90 [0.71–1.15];
	A/Brisbane/59/2007-like	20	7/51 (13.7)	*p* = 0.39*	*p* = 0.40*
		40	2/19 (10.5)		
		≥80	0/14 (0.0)		
2	H3N2	<=10	67/404 (16.6)	0.69 (0.52–0.90);	0.61 [0.44–0.84];
	A/Perth/16/2009-like	20	2/48 (4.2)	*p* = 0.008	*p* = 0.002
		40	2/23 (8.7)		
		≥80	0/19 (0.0)		
2	B/Florida/04/2006-like	<=10	47/302 (15.6)	0.79 [0.65–0.97];	0.78 [0.63–0.97];
		20	6/93 (6.5)	*p* = 0.03	*p* = 0.02
		40	4/73 (5.5)		
		≥80	2/26 (7.7)		
3	H1N1	<=10	103/526 (19.6)	0.54 [0.27–1.10];	0.77 [0.40–1.49];
	A/California/04/09-like	≥20	1/14 (7.1)	*p* = 0.09	*p* = 0.43

Note that titers are modeled as a continuous variable and do not use the categorization shown, therefore 0 values in a category do not prevent analysis. */** Test for non-linear (quadratic) effect of (log2-) titer on outcome: *p* < 0.05 (*). Between-strain comparisons of titer effects on the risk of infection (not adjusted for age): - H1 vs. H3: *p* = 0.07 (using data from all 3 seasons) [*p* = 0.87 (season 1), *p* = 0.008 (season 2)]. - H1 vs. B: *p* = 0.003 (using data from all 3 seasons) [*p* = 0.10 (season 1), *p* = 0.03 (season 2)]. Between-strain comparisons of titer effects on the risk of infection (adjusted for age): - H1 vs. H3: *p* = 0.03 (using data from all 3 seasons) [*p* = 0.82 (season 1), *p* = 0.006 (season 2)]. - H1 vs. B: *p* = 0.02 (using data from all 3 seasons) [*p* = 0.18 (season 1), *p* = 0.09 (season 2)].

**Table 3:** Effect of previous infection with a different strain on current infection, adjusted for pre-season HI titer and age.

Outcome	Variable	*n* infected/included (%)	OR for prior infection [95% CI]
Season 2 –	H3 season 1		
H1N1	– not infected	47/482 (9.8)	0.18 [0.01–3.09]; *p* = 0.24^a^
A/Brisbane/59/2007	– infected	0/12 (0.0)	
	B season 1		
	– not infected	41/429 (9.6)	0.89 [0.32–2.43]; *p* = 0.81
	– infected	6/65 (9.2)	
Season 2 –	H1N1 season 1		
H3N2	– not infected	68/460 (14.8)	0.48 [0.15–1.55]; *p* = 0.22
A/Perth/16/2009^b^	– infected	3/34 (8.8)	
	B season 1		
	– not infected	61/429 (14.2)	1.08 [0.52–2.22]; *p* = 0.84
	– infected	10/65 (15.4)	
Season 2 –	H1 season 1		
B/Florida/04/2006^b^	– not infected	57/460 (12.4)	0.39 [0.09–1.73]; *p* = 0.22
	– infected	2/34 (5.9)	
	H3 season 1		
	– not infected	59/482 (12.2)	0.16 [0.01–2.75]; *p* = 0.21^a^
	– infected	0/12 (0.0)	
Season 3 –	H1 season 2		
H1N1	– not infected	97/491 (19.8)	0.23 [0.08–0.62]; *p* = 0.004^c^
A/California/04/09	– infected	5/49 (10.2)	
	H3 season 2		
	– not infected	87/469 (18.6)	0.88 [0.45–1.74]; *p* = 0.72
	– infected	15/71 (21.1)	
	B season 2		
	– not infected	85/483 (17.6)	2.40 [1.14–5.05]; *p* = 0.02^c^
	– infected	17/57 (29.8)	

a Based on Bayesian logistic regression and ignoring potential household clustering to cope with separation.

b Pre-season titers remain significant for S2–H3 and S2–B after adjusting for prior infection with a different serotype (and age): all *p* < 0.05.

c Both *p*-values remain significant if H1 season 2 and B season 2 are included jointly into the logistic model (*p* = 0.002 for H1 season 2, *p* = 0.01 for B season 2). *p*-values for analysis without adjustment for age and pre-season titer: 0.11 (H1 season 2) and 0.05 (B season 2).
