# Supplementary material for: Hemagglutination inhibiting antibodies and protection against seasonal and pandemic influenza infection
Source: J Infect. 2015 Feb;70(2):187–96. doi: 10.1016/j.jinf.2014.09.003 (PMC4309889; doi:10.1016/j.jinf.2014.09.003)
Supplement: Supplementary file 1 [file mmc1.docx]

|  | | **Ferret anti-sera against viruses shown below ---->** | | | | | | | |
| --- | --- | --- | --- | --- | --- | --- | --- | --- | --- |
| **H3N2** | |  | |  | | |  | | |
| Clade | | CA2004 | | BR2007 | | | PE2009 | | |
| Strain | | Singapore/37/04 | Brisbane/3/05 | Brisbane/10/07 | Uruguay/716/07 | Perth/27/07 | Victoria/208/09 | Perth/16/09 | Victoria/8/10 |
| **isolates** | |  |  |  |  |  |  |  |  |
| 2008 | EL134 | 160 | <10 | **640** | **640** | 320 | 80 | 80 | 640 |
|  | EL135 | 80 | <10 | **640** | 320 | **640** | 320 | 320 | 640 |
|  | EL140 | 80 | <10 | **640** | **640** | 320 | 160 | 40 | 320 |
| 2009 | EL196 | 80 | <10 | **320** | **320** | **320** | 160 | 80 | **320** |
|  | EL201 | <10 | <10 | 80 | 40 | 20 | 160 | 320 | **640** |
|  | EL204 | <10 | <10 | 80 | 40 | 40 | 1280 | 1280 | **2560** |
|  | EL206 | <10 | <10 | 80 | <10 | <10 | 320 | **640** | **640** |
| Reference strain | | **320** | **640** | **640** | **320** | **640** | **320** | **160** | **640** |
| **B Yamagata** | |  |  |  |  |  |  |  |  |
|  | | 2006 | 2007 |  | 2008 |  |  | 2009 | 2012 |
|  |  | Florida/4/06 | Brisbane/3/07 | Bang/3333/07 | SthAust/5/08 | Indiana/1/08 | Brisbane/9/08 | Hubei/158/09 | WELL/3/12 |
| 2008 | EL111 | **640** | **640** | 80 | 80 | 160 | 80 | 160 | **640** |
|  | EL120 | **640** | **640** | 80 | 80 | 160 | 160 | 160 | **640** |
|  | EL119 | **320** | **320** | 40 | 40 | 80 | 80 | 80 | **320** |
|  | EL156 | **640** | **640** | 80 | 80 | 160 | 80 | 160 | **640** |
|  | EL162 | **640** | **640** | 40 | 40 | 160 | 80 | 80 | **640** |
|  | EL110 | **640** | **640** | 80 | 80 | 160 | 80 | 160 | **640** |
| Reference strain | | **>2560** | **1280** | **1280** | **320** | **640** | **320** | **640** | **1280** |
| **B Victoria** | |  |  |  |  |  |  |  |  |
|  |  | 2004 | 2008 |  |  |  | 2009 | 2010 | 2011 |
|  |  | Mal/2506/04 | Singapore/616/08 | Brisbane/60/08 | Brisbane/33/08 | HK/90/08 | PHIL/6363/09 | SYD/508/10 | CAMB/30/11 |
| 2009 | EL189 | 160 | 320 | 640 | 160 | 160 | 160 | 160 | **>2560** |
| Reference strain | | **640** | **640** | **320** | **>2560** | **1280** | **320** | **>2560** | **>2560** |

Supplementary Table S1: Antigenic characterization of viruses isolated from cohort participants in S1 and S2 using ferret anti-sera against reference strains

Supplementary Table S2. Pre and post-season HI antibody titers in participants with RT-PCR confirmed infection

|  |  |  | reciprocal HI titer | | |  |
| --- | --- | --- | --- | --- | --- | --- |
| Season | Subtype | Seroconvert | pre-season | post-season | ratio | symptomatic |
| 1 | H1N1 | + | 5 | 80 | 16 | yes |
| 1 | H1N1 | + | 5 | 40 | 8 | yes |
| 1 | H1N1 | + | 5 | 40 | 8 | yes |
| 1 | H1N1 | + | 5 | 40 | 8 | yes |
| 1 | H1N1 | - | 5 | 20 | **4** | yes |
| 1 | H1N1 | - | 5 | 20 | **4** | yes |
| 1 | H1N1 | - | 40 | 80 | **2** | yes |
| 1 | H1N1 | - | 5 | 10 | **2** | yes |
| 1 | H1N1 | - | 5 | 5 | 1 | yes |
| 1 | H3N2 | + | 40 | 160 | 4 | yes |
| 1 | H3N2 | + | 10 | 80 | 8 | yes |
| 1 | H3N2 | + | 10 | 160 | 16 | yes |
| 2 | H1N1 | + | 5 | 160 | 32 | Yes |
| 2 | H1N1 | + | 20 | 640 | 32 | Yes |
| 2 | H1N1 | - | 5 | 5 | 1 | Yes |
| 2 | H3N2 | + | 5 | 640 | 128 | Yes |
| 2 | H3N2 | + | 5 | 80 | 16 | yes |
| 2 | H3N2 | + | 5 | 160 | 32 | yes |
| 2 | H3N2 | + | 10 | 640 | 64 | yes |
| 2 | H3N2 | + | 10 | 640 | 64 | yes |
| 2 | H3N2 | + | 5 | 160 | 32 | yes |
| 3 | H1N1 | + | 5 | 40 | 8 | yes |
| 3 | H1N1 | + | 5 | 80 | 16 | yes |
| 3 | H1N1 | + | 5 | 160 | 32 | yes |
| 3 | H1N1 | + | 5 | 160 | 32 | yes |
| 3 | H1N1 | + | 5 | 40 | 8 | yes |
| 3 | H1N1 | + | 5 | 40 | 8 | yes |
| 3 | H1N1 | + | 5 | 160 | 32 | yes |
| 3 | H1N1 | + | 5 | 40 | 8 | yes |
| 3 | H1N1 | + | 5 | 40 | 8 | yes |
| 3 | H1N1 | + | 5 | 40 | 8 | yes |
| 3 | H1N1 | + | 5 | 80 | 16 | yes |
| 3 | H1N1 | + | 5 | 40 | 8 | yes |
| 3 | H1N1 | + | 5 | 320 | 64 | no |
| 3 | H1N1 | + | 5 | 160 | 32 | no |
| 3 | H1N1 | + | 5 | 40 | 8 | no |
| 3 | H1N1 | + | 5 | 40 | 8 | no |
| 3 | H1N1 | + | 5 | 320 | 64 | no |
| 3 | H1N1 | - | 5 | 5 | 1 | yes |
| 3 | H1N1 | - | 5 | 5 | 1 | yes |
| 3 | H1N1 | - | 5 | 20 | **4** | yes |
| 3 | H1N1 | - | 5 | 5 | 1 | yes |
| 3 | H1N1 | - | 5 | 10 | **2** | no |
| 3 | H1N1 | - | 5 | 10 | **2** | no |

Supplementary Table S3. Pre-season HI titer detection and effect on homosubtypic infection defined as RT-PCR confirmed or 4-fold rise in titer

| **Season** | **Infecting strain** | **Pre-season HI titer** | **n infected / n with titer (%)** | **OR for each 2-fold titer increase [95% CI]** | **OR for each 2-fold titer increase, adjusted for age [95% CI]** |
| --- | --- | --- | --- | --- | --- |
| 1 | H1N1 | <10 | 49/375 (13.1) | 0.69 [0.43-1.10] | 0.63 [0.38-1.03] |
|  | A/Brisbane/59/2007-like | 10 | 6/111 (5.4) | p = 0.12 | p = 0.07 |
|  |  | 20 | 3/36 (8.3) |  |  |
|  |  | 40 | 2/21 (9.5) |  |  |
|  |  | 80 | 0/5 (0.0) |  |  |
| 1 | H3N2 | <10 | 17/328 (5.2) | 0.69 [0.50-0.94] | 0.59 [0.39-0.90] |
|  |  | 10 | 3/31 (9.7) | p = 0.02 | p 0.01 |
|  | A/Brisbane/10/2007-like | 20 | 1/73 (1.4) |  |  |
|  |  | 40 | 2/56 (3.6) |  |  |
|  |  | ≥80 | 0/60 (0.0) |  |  |
| 1 | B/Florida/04/2006-like | <10 | 52/135 (38.2) | 0.46 [0.35-0.59] | 0.45 [0.35-0.59] |
|  |  | 10 | 28/150 (18.8) | p < 0.001 | p < 0.001 |
|  |  | 20 | 8/121 (6.6) |  |  |
|  |  | 40 | 8/67 (11.9) |  |  |
|  |  | ≥80 | 1/75 (1.3) |  |  |
| 2 | H1N1 | <10 | 48/354 (13.6) | 0.89 [0.71-1.11] | 0.74 [0.58-0.95] |
|  | A/Brisbane/59/2007-like | 10 | 11/56 (19.6) | p = 0.30 | p = 0.02 |
|  |  | 20 | 7/51 (13.7) |  |  |
|  |  | 40 | 2/19 (10.5) |  |  |
|  |  | ≥80 | 0/14 (0.0) |  |  |
| 2 | H3N2 | <10 | 84/321 (26.2) | 0.51 [0.37-0.70] | 0.44 [0.30-0.63] |
|  | A/Perth/16/2009-like | 10 | 16/83 (19.3) | p < 0.001 | p < 0.001 |
|  |  | 20 | 2/48 (4.2) |  |  |
|  |  | 40 | 2/23 (8.7) |  |  |
|  |  | ≥80 | 0/19 (0.0) |  |  |
| 2 | B/Florida/04/2006-like | <10 | 57/195 (29.2) | 0.54 [0.42-0.69] | 0.52 [0.40-0.68] |
|  |  | 10 | 24/107 (22.4) | p < 0.001 | p < 0.001 |
|  |  | 20 | 6/93 (6.4) |  |  |
|  |  | 40 | 4/73 (5.5) |  |  |
|  |  | ≥80 | 2/26 (7.7) |  |  |
| 3 | H1N1 | <10 | 128/506 (25.2) | 0.70 [0.41-1.20] | 0.91 (0.54-1.52) |
|  | A/California/04/09-like | 10 | 5/20 (25.0) | p = 0.20 | p = 0.71 |
|  |  | 20 | 0/9 (0.0) |  |  |
|  |  | 40 | 1/3 (33.0) |  |  |
|  |  | ≥80 | 0/2 (0.0) |  |  |

Notes:

- Any 4-fold rise in titer was considered as infection including titer rises from 5 to 20.
- There was no evidence for a non-linear (quadratic) effect of (log2-)titer on outcome (all p>0.05) except for the unadjusted analysis for H1S2 (p=0.047).
- There was also no evidence for an interaction between titer and age (all p>0.35).

Between-strain comparisons of titer effects on the risk of infection (not adjusted for age):

- H1 vs H3: p=0.04 (using data from all 3 seasons) [p=0.30 (season 1), p=<0.001 (season 2)]
- H1 vs B: p<0.001 (using data from all 3 seasons) [p=0.002 (season 1), p<0.001 (season 2)]

Between-strain comparisons of titer effects on the risk of infection (adjusted for age):

- H1 vs H3: p=0.02 (using data from all 3 seasons) [p=0.33 (season 1), p<0.001 (season 2)]
- H1 vs B: p=0.002 (using data from all 3 seasons) [p=0.004 (season 1), p<0.001 (season 2)]
